# Properties of Polybenzoxazine-Based Conducting Materials in Energy-Related Applications

**DOI:** 10.3390/polym17162194

**Published:** 2025-08-11

**Authors:** Shakila Parveen Asrafali, Thirukumaran Periyasamy, Gazi A. K. M. Rafiqul Bari, Jaewoong Lee

**Affiliations:** 1 Department of Fiber System Engineering, Yeungnam University, Gyeongsan 38541, Republic of Korea; shakilaparveen@yu.ac.kr (S.P.A.); thirukumaran@ynu.ac.kr (T.P.); 2 Department of Mechanical Engineering, Gachon University, Seongnam-si 13120, Republic of Korea

**Keywords:** conducting polymers, polybenzoxazine, structure–property relationship, energy storage applications

## Abstract

Polybenzoxazine (PBz)-based conducting materials have gained significant attention due to their unique combination of thermal stability, mechanical strength, and electrical conductivity. These polymers integrate the inherent advantages of polybenzoxazines—such as low water absorption, high glass transition temperature, and excellent chemical resistance—with the electrical properties of conducting polymers like polyaniline, polypyrrole, and polythiophene. The incorporation of conductive elements in polybenzoxazine networks can be achieved through blending, in situ polymerization, or hybridization with nanostructures such as graphene, carbon nanotubes, or metallic nanoparticles. These modifications enhance their charge transport properties, making them suitable for applications in flexible electronics, energy storage devices, sensors, and electromagnetic shielding materials. Furthermore, studies highlight that polybenzoxazine matrices can improve the processability and environmental stability of conventional conducting polymers while maintaining high conductivity. The structure–property relationships of polybenzoxazine-based composites demonstrate that tailoring monomer composition and polymerization conditions can significantly influence their conductivity, thermal stability, and mechanical properties. This review summarizes recent advancements in PBz composites, focusing on their synthesis, structural modifications, conductivity mechanisms, and potential applications in advanced energy storage systems.

## 1. Introduction

Benzoxazine resins represent a new class of nitrogen-containing thermosetting resins characterized by numerous advantageous properties such as high char yield, excellent thermal stability, near-zero volumetric shrinkage during polymerization, low water absorption, high glass transition temperature, good molecular design flexibility, and compatibility with other polymers. These resins polymerize into a three-dimensional polybenzoxazine (PBZ) network via ring-opening polymerization of benzoxazine monomers without requiring any hardeners or catalysts [1,2,3,4,5]. Bifunctional benzoxazines, synthesized from bisphenols or monophenols, monoamines or diamines, and formaldehyde, demonstrate superior performance compared to monofunctional counterparts due to higher crosslinking density and enhanced thermal and mechanical stability. Polybenzoxazine is also noted as a non-fluorinated, non-silicone, low surface free-energy polymeric material that is more cost-effective and easier to process than fluoropolymers. Despite these strengths, limitations such as high curing temperatures and brittleness have hindered widespread application [6,7,8,9,10,11]. To address these issues, researchers have employed strategies like introducing flexible chains, blending with elastomers or plastics, and utilizing reaction-induced phase separation to improve toughness. Additionally, catalytic systems involving primary amines, imidazoles, organic acids, and metal halides have been developed to lower the curing temperature [12,13,14]. The unique chemistry of benzoxazines enables a range of processing advantages, including low melt viscosity, no volatile release during curing, and design flexibility without requiring harsh catalysts. As high-performance thermosets, polybenzoxazines not only form highly crosslinked networks upon thermal treatment but also serve as excellent precursors for nitrogen-doped carbon due to their near-zero shrinkage and high residual char yield during pyrolysis. Furthermore, with C, N, and O atoms in their molecular structure, they offer an ideal platform for tuning photocatalytic properties in advanced materials applications [15,16,17,18,19,20,21].

### 1.1. Various Energy-Related Applications and Their Key Challenges

Supercapacitors are widely explored for their high-power density, fast charge/discharge rates, long cycle life, and safety. Carbon-based materials are popular for EDLCs due to their high surface area and stability, but suffer from low capacitance and energy density [22,23,24,25,26]. To improve performance, hybrid electrodes combining carbon with pseudocapacitive materials like conducting polymers or metal oxides are used. Incorporating nanostructures (e.g., CNTs, GO, RGO) enhances conductivity and ion transport [27,28,29,30,31,32]. Flexible, strong electrode designs are essential. Though carbon is easy to prepare, its powder form complicates processing; organic polymers offer eco-friendly, efficient alternatives. To reduce fossil fuel reliance, research focuses on electrochemical systems like water splitting. Pt-based HER catalysts are efficient but costly, leading to interest in non-Pt alternatives, especially nitrogen-doped carbon, which offers good conductivity, stability, and activity for HER, ORR, and OER [33,34,35,36,37]. Recent progress includes metal-encapsulated and heteroatom-doped carbon composites. N-doped carbon enables bifunctional activity due to its tunable structure and low cost [38,39,40]. Fuel cells, particularly PEMFCs, are promising clean energy devices with high power density, long life, and low emissions. They require components with high conductivity, strength, and water resistance [41,42,43]. Nafion, the standard membrane, offers high proton conductivity but has drawbacks like water management and fuel crossover, limiting broader use. Heteroatom-doped porous organic polymers (POPs) are promising for CO_2_ capture due to their tunable pore structures and strong CO_2_ binding via interactions with electron-rich heteroatoms. POPs like CTFs, CMPs, and HCPs offer high uptake capacity and are effective, low-energy sorbents for carbon capture and storage [44,45,46,47,48].

### 1.2. Materials Used in Combination with Polybenzoxazine

Graphene, composed of a one-atom-thick sheet of sp^2^-bonded carbon atoms arranged in a two-dimensional honeycomb lattice, boasts exceptional properties such as high theoretical surface area, excellent electrical conductivity, and strong chemical stability. However, its practical application is hindered by issues like aggregation and restacking due to van der Waals forces, which reduce its electrochemical performance and processability [49,50]. To address this, graphene oxide (GO), with its abundant oxygen-containing functional groups, offers improved dispersion in water and organic solvents, making it more suitable for composite fabrication. Ketjen black, a highly conductive carbon with a large specific surface area and branched morphology, is also widely employed in electrochemical capacitors and fuel cells as a conductive support. In parallel, polymeric ionic liquids (PILs), particularly hyperbranched PILs (HPILs), have gained significant attention due to their multifunctional architecture, high thermal and mechanical stability, and excellent processability [51,52]. These properties make HPILs ideal for applications ranging from catalysis and electrochemical devices to wastewater treatment and nanoparticle modification. Conductive polymers like polyaniline (PANI) are also notable for their easy synthesis, eco-friendliness, and high theoretical capacity; however, their performance suffers from structural degradation during repeated doping and de-doping cycles, leading to low cycling stability. Meanwhile, sulfonated hydrocarbon polymers, such as sulfonated polybenzimidazoles (PBIs), poly(arylene ethers), and polyimides, are being explored as alternatives to Nafion in polymer electrolyte membranes due to their low cost, chemical/thermal stability, and ion-conductive structures [53,54,55]. Nevertheless, their long-term chemical durability is limited by structural vulnerabilities, particularly the acid functional groups and ether linkages that are prone to nucleophilic attack. Together, these materials and innovations contribute to the evolving landscape of electrochemical energy storage and conversion systems.

### 1.3. Role of Hetero Atom in PBz/PBz Derived Carbon

Incorporating heteroatoms, particularly nitrogen, into carbon frameworks significantly enhances their electrochemical performance by increasing surface polarity, wettability, and electrical conductivity, and introducing pseudocapacitance through reversible Faradaic reactions [23,24]. Nitrogen-enriched porous carbons (NCs), often synthesized from nitrogen-rich precursors, offer a scalable route to high-performance materials. Nitrogen-rich PBz enhances interfacial electronic coupling with carbon-based materials through π–π stacking and hydrogen bonding. Its aromatic and nitrogen-functional groups align with sp^2^-carbon frameworks like graphene or carbon nanotubes, promoting orbital overlap and charge transfer. DFT studies confirm adsorption energies (around −0.7 to −1.2 eV) and hydrogen bond formation (~2.8 Å), supporting modest charge transfer (0.1–0.3 e^−^). These interactions improve interfacial charge delocalization and adhesion, boosting electronic performance in applications such as sensing, catalysis, and energy storage [56,57]. Ordered mesoporous carbons (OMCs) and nitrogen-doped variants are especially attractive for energy applications due to their tunable pore structure, high surface area, and improved charge transport. Common synthesis methods, such as hard-template, EISA, and post-synthesis doping, allow structural control but often face challenges in scalability and cost [58]. To overcome limitations of conventional microporous carbons, template-assisted methods using agents like colloidal silica, SBA-15, or polystyrene beads are employed to create well-ordered, interconnected pores suitable for harsh environments [59,60,61,62]. Porous organic polymers (POPs), composed of light elements such as C, N, O, and B, offer additional tunability in porosity and functionality. Hybrid materials—combining heteroatom-doped metal compounds with nitrogen-doped carbon matrices—show bifunctional activity for both ORR and OER. Doping with elements like N, P, or B alters the electronic structure and enhances conductivity, while carbon shells prevent agglomeration and corrosion of metal nanoparticles. These materials provide a cost-effective and scalable alternative to noble metal catalysts for energy conversion and storage applications.

The objective of this review is to provide a comprehensive overview of recent advancements in polybenzoxazine-based conducting materials, with a particular focus on their synthesis strategies, structural modifications, and structure–property relationships. The review aims to elucidate how the unique integration of polybenzoxazine matrices with conductive elements, such as polyaniline, carbon nanostructures, and metallic nanoparticles, enhances the electrical, mechanical, and thermal properties of the resulting hybrid materials. Emphasis is placed on understanding the mechanisms of conductivity and how tailored monomer compositions and polymerization conditions influence performance. Ultimately, the review highlights the potential of PBz composites in next-generation energy storage applications, including supercapacitors, batteries, and flexible electronics.

## 2. Incorporating Conductivity into PBz

**Figure 1:**
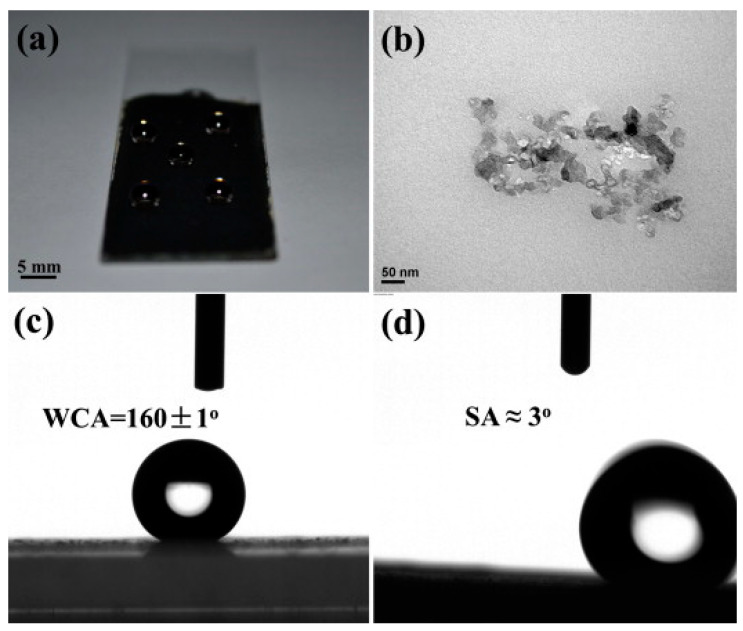
(**a**) Optical image of water droplets placed on a KB-PBZ composite coating, (**b**) TEM image of Ketjen black, (**c**) water contact angle, and (**d**) sliding angle of KB-PBZ composite coating. Reprinted with permission from Reference [6]. Copyright 2013 Elsevier.

Shen et al. (2013) [6] demonstrated a straightforward drop-casting method to fabricate superhydrophobic and conductive Ketjen black–polybenzoxazine (KB-PBZ) composite coatings. A mixture of benzoxazine monomer (BA-a) and Ketjen black in tetrahydrofuran was drop-cast onto various substrates and thermally cured. The Ketjen black not only contributed to electrical conductivity but also played a critical role in surface roughness, promoting superhydrophobicity (WCA ≈ 160°, SA ≈ 3°), as shown in Figure 1. Shen et al. also showed that the roughness induced by Ketjen black aggregation created hierarchical structures that trap air and reduce the contact area with water, enabling persistent superhydrophobicity over a wide pH range and thermal conditions. Careful control of synthesis parameters, including monomer structure, filler type and loading, and curing conditions directly influences the electronic, thermal, and surface properties of PBz-based conducting polymers, tailoring them for diverse applications such as energy storage, sensors, and protective coatings.

**Figure 2:**
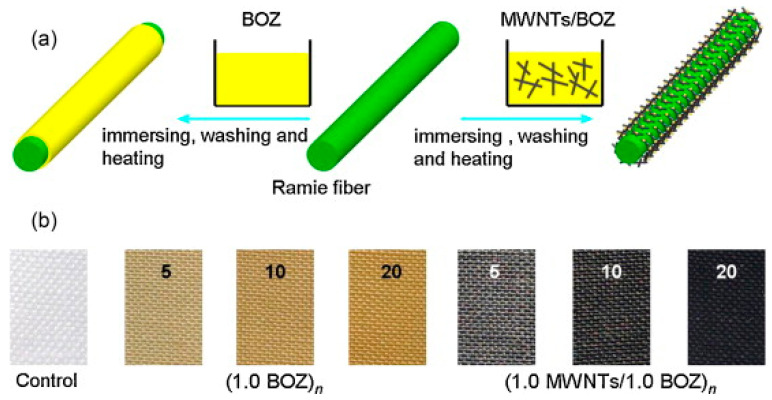
(**a**) Schematic illustration for the construction process of poly(BOZ) and MWNTs/poly(BOZ) nanocomposites on ramie fabric and (**b**) Pictures of the pristine ramie fabric (control), (1.0 BOZ)*_n_* and (1.0 MWNTs/1.0 BOZ)*_n_* systems. Reprinted with permission from Reference [63]. Copyright 2014 Elsevier.

Zhang et al. (2014) [63] developed a simple and effective solution-immersion process to fabricate MWNTs/polybenzoxazine nanocomposite coatings on ramie fabric. The process involved (i) preparing a stable dispersion of multiwalled carbon nanotubes (MWNTs) in dimethylformamide (DMF), (ii) mixing the MWNTs with a synthesized benzoxazine monomer (BOZ), (iii) repeated immersion cycles of ramie fabric into this MWNTs/BOZ mixture, and (iv) thermal curing at 130 °C to partially polymerize the benzoxazine monomer, represented in Figure 2. The number of immersion cycles and the MWNT concentration were systematically varied to tune surface loading and coating thickness. The resulting nanocomposite coating exhibited a hierarchical 3D interpenetrating network, which was responsible for dual functional performance, i.e., superhydrophobicity and electrical conductivity. An increased number of immersion cycles and higher MWNT concentration resulted in enhanced surface roughness and reduced surface energy, yielding water contact angles (WCA) up to 152° and sliding angles (SA) as low as 3°. A denser MWNT network led to significantly improved conductivity, with sheet resistance dropping to 3.41 × 10^3^ Ω/sq after 20 cycles at 1.0 mg/mL MWNT concentration. The improved hydrophobicity was primarily due to both the low surface energy of partially cured polybenzoxazine and the nanostructured roughness introduced by MWNTs [64,65,66]. Meanwhile, conductivity was enhanced by the formation of percolation pathways across the nanocomposite coating.

**Figure 3:**
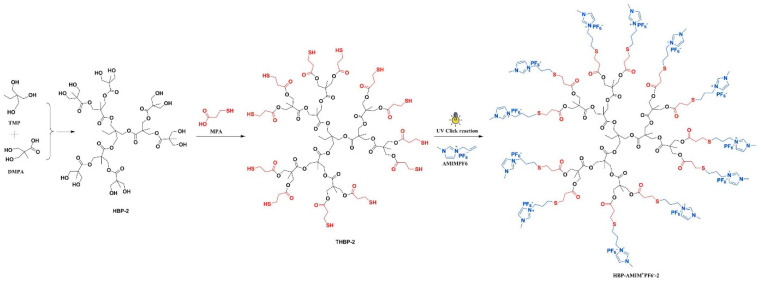
Synthesis of hyperbranched polyester ionic liquids. Reprinted with permission from Reference [8]. Copyright 2018 Elsevier.

Chen et al. (2018) [8] synthesized a novel hyperbranched polymeric ionic liquid (HBP-AMIM^+^PF_6_^−^) using a two-step process: (i) esterification and (ii) Thiol-ene click reaction. Thiol-ended hyperbranched polyesters (THBPs) were synthesized by reacting hydroxyl-ended hyperbranched polyesters with 3-mercaptopropionic acid. The THBPs were reacted with 1-allyl-3-methylimidazolium hexafluorophosphate (AMIMPF_6_) under UV light, yielding HBP-AMIM^+^PF_6_^−^. This HBP-AMIM^+^PF_6_^−^ was then blended into benzoxazine/epoxy (BA/ECC) systems and thermally cured via a multi-step heating process (120 °C to 200 °C) (Figure 3). The ionic liquid acted both as a reactive additive and a catalytic agent for curing. The integration of HBP-AMIM^+^PF_6_^−^ into the BA/ECC matrix resulted in significant improvements in processing and performance.

The ionic liquid drastically reduced the curing temperature and gel time by catalyzing the ring-opening polymerization of benzoxazine. The onset and peak-curing temperatures dropped by up to 40.2 °C and 23.4 °C, respectively, for 7 wt.% loading. With respect to mechanical properties, such as an optimal loading of 3 wt.%, the composite exhibited tensile strength increased by 76.6%; tensile toughness increased by 279.3%; flexural strength increased by 57.8%; flexural modulus increased by 22.3%; and impact strength increased by 80.4%. These enhancements were attributed to in situ toughening from hyperbranched structures that absorb impact energy, increased crosslinking density due to multiple reactive terminal imidazolium groups, and homogeneous microstructure, as confirmed by SEM, with no phase separation [67,68]. Additionally, improved thermal stability was observed, with the decomposition temperature (T_5_%) increasing by over 30 °C at optimal HBP-AMIM^+^PF_6_^−^ loadings. Dynamic mechanical analysis showed increased storage modulus and glass transition temperature (T_g_), indicating stronger and stiffer networks. Overall, the structure of the hyperbranched ionic liquid, featuring multiple functional groups, internal free volume, and ionic segments, played a pivotal role in enhancing the mechanical toughness, thermal stability, and processability of the PBZ-based thermosets.

## 3. Intriguing Properties of PBz and Their Composites

Sirawit et al. (2020) [41] synthesized highly filled polybenzoxazine (PBA) composites for use as bipolar plates in proton exchange membrane fuel cells (PEMFCs). Benzoxazine monomer (BA-a) was synthesized using bisphenol-A, formaldehyde, and aniline via a solvent-free route. A mixture of benzoxazine resin with a constant amount of graphene (7.5 wt.%) and varying ratios of graphite and multiwalled carbon nanotubes (CNTs) (totaling 84 wt.% filler) was prepared using melt mixing at 90–100 °C for 30–45 min. The composite mixture was molded using compression molding at 200 °C and 150 MPa for 3 h to achieve full crosslinking. The performance of the resulting composites was deeply influenced by the filler content and dispersion of the filler. The addition of CNTs significantly improved the thermal conductivity, reaching 21.3 W/m·K at 2 wt.% CNTs. This enhancement is due to the 3D conductive network formed by the high aspect ratio and bridging effect of CNTs between graphite and graphene particles. With increasing CNT content, in-plane electrical conductivity improved, peaking at 364 S/cm at 2 wt.% CNTs. This is attributed to the continuous conductive pathways enabled by overlapping CNT networks [69,70]. The flexural strength and modulus were also enhanced with CNT addition. At 2 wt.% CNTs loading, flexural strength reached 41.5 MPa and flexural modulus reached 49.7 GPa. Despite higher CNT content, water uptake remained low (<0.114% after 24 h) due to good matrix-filler adhesion and the low polarity of PBA. SEM and TEM images showed well-dispersed CNTs, graphene, and graphite in the polymer matrix. The low melt viscosity of PBA facilitated uniform dispersion and strong filler-matrix interfacial bonding.

**Figure 4:**
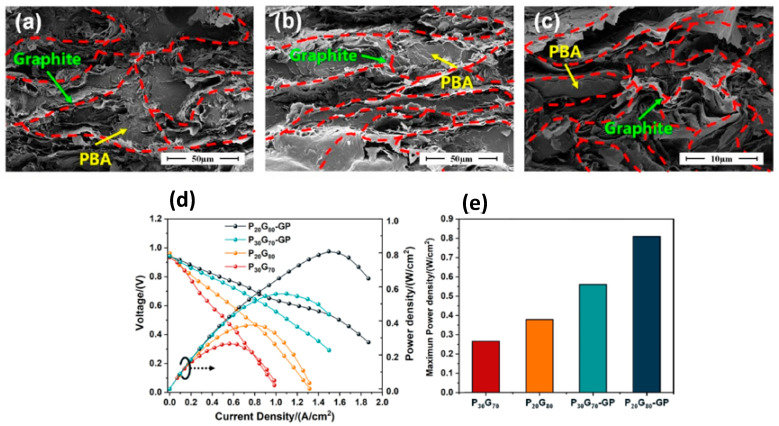
SEM images of different BPs’ internal sections: (**a**) P_60_G_40_, (**b**) P_40_G_60_, (**c**) P_20_G_80_; (**d**) performance combo chart; and (**e**) maximum power density of PEMFC single cell with composite BP P_30_G_70_, P_20_G_80_, P_30_G_70_-GP, and P_20_G_80_-GP. Reprinted with permission from Reference [71]. Copyright 2023 Elsevier.

Huang et al. (2023) [71] fabricated a high-performance polybenzoxazine/expanded graphite (PBA/EG) composite bipolar plate (BP) with a multilayer ‘graphite–composite–graphite’ structure. At first, benzoxazine (PBA), expanded graphite (EG), and expansible graphite powders were dried to eliminate moisture. And then, PBA and EG were blended at high speed (25,000 rpm) to create a uniform mixture with specific weight ratios (e.g., P30G70 = 30 wt.% PBA, 70 wt.% EG). Finally, expansible graphite was heat-treated at 800 °C and pressed into thin sheets to form graphite paper (GP). The composite mixture was sandwiched between two GP layers and compression-molded at 180 °C under 25 MPa pressure for 20 min to yield composite BPs with integrated conductive networks and improved surface contact layers. The multilayer design significantly enhanced the mechanical, electrical, and thermal performance of the composite BPs. In-plane conductivity reached 278.85 S/cm, and area-specific resistance (ASR) dropped to 9.70 mΩ·cm^2^. The EG formed an interconnected conductive network, while the graphite surface layers minimized contact resistance and avoided resin enrichment, creating efficient charge transfer pathways, as depicted in Figure 4 [72,73]. Even with high graphite content (up to 80 wt.%), the composite maintained a flexural strength of 75.75 MPa. PBA acted as a binding matrix, enhancing mechanical integrity without compromising electrical performance. Thermal conductivity was enhanced by up to 36.2% due to the addition of graphite layers, aiding in heat dissipation during PEMFC operation. Hydrophobicity improved with increased graphite content, as could be observed with water contact angles, which were consistently around 99–102°, ensuring effective water removal in fuel cell environments. Corrosion current densities remained below 1 mA/cm^2^, even at high graphite loadings. The power density of single PEMFC cells increased by over 110% when using the multilayer BP structure compared to conventional configurations.

**Figure 5:**
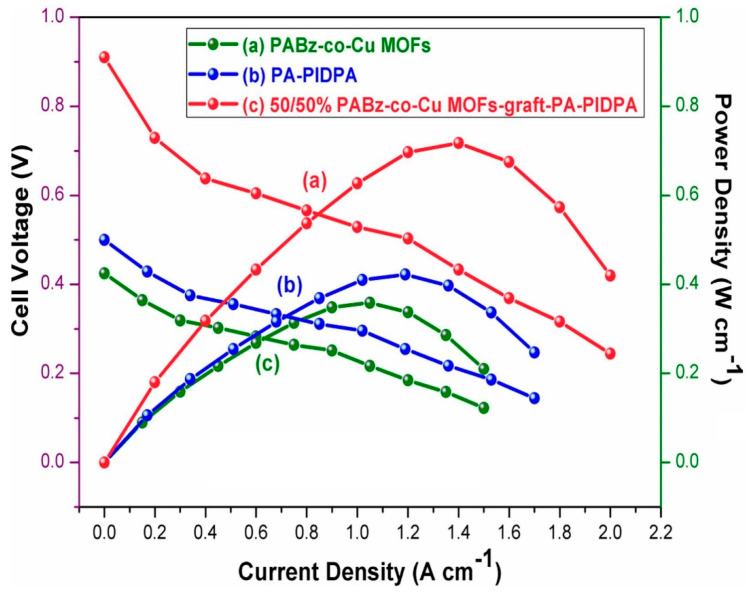
Fuel cell performance of 50/50% combination of PABz-co-Cu MOFs-graft-PIDPA, PA-PIDPA, and PABz-co-Cu MOFs at 150 °C without RH. Reprinted with permission from Reference [53]. Copyright 2024 Elsevier.

Murugan et al. (2024) [53] developed a novel aryl ether-free, covalently crosslinked polymer membrane aimed at high-performance applications in HT-PEMFCs and supercapacitors. The synthesis involved (i) preparation of PIDPA (poly(imidazole-co-diphenylamine)) via oxidative polymerization using imidazole, diphenylamine, and FeCl_3_, (ii) synthesis of MFBz monomer through a solvent-free mechano-chemical grinding of ethylene diamine, paraformaldehyde, and 5-sulphosalicylic acid, (iii) formation of ABz-co-Cu MOFs using DL-aspartic acid and MFBz coordinated with Cu^2+^ ions through interfacial polymerization, (iv) thermal curing of PIDPA and ABz-co-Cu MOFs using a stepwise heating protocol (80–220 °C), yielding thin-film membranes, and (v) doping with phosphoric acid to enhance the proton conductivity of the membranes. The integration of PA-doped PIDPA and Cu-MOF bridged benzoxazine created a robust, highly functional polymer with improved properties. The optimal 50/50 wt.% membrane showed the highest proton conductivity (PC) of 0.0757 S/cm at 200 °C, more than doubling the PC of the individual components. This improvement is due to a synergistic hydrogen-bonded network formed by imidazole/amine groups and sulfonic acids, facilitating fast proton transport [74]. Moreover, the 50/50 membrane displayed a tensile strength of 3.87 MPa and elongation at break of 23.93%, due to the dense three-dimensional crosslinked network and hydrogen bonding interactions. Increased crosslink density improved rigidity, while still retaining flexibility. TGA analysis showed thermal stability beyond 700 °C, and oxidative stability was retained up to 90.1% weight after exposure to Fenton’s reagent, due to both the network architecture and Cu_2_O’s radical scavenging effect. PIDPA, a conjugated polymer with imidazole and diphenylamine moieties, is a known electroactive species, capable of redox transitions during supercapacitor operation. When crosslinked with PBO-Cu-MOFs, the redox-active PIDPA gains improved mechanical and thermal stability from the polymer network, resulting in enhanced specific capacitance, higher proton conductivity, and greater oxidative resistance. The redox behavior in the system arises from the acid–base interaction and hydrogen bonding between phosphoric acid, imidazole, and amine groups in PIDPA and sulfonic acid in the PBO framework. With respect to electrochemical performance, the 50/50 membrane achieved a peak power density of 0.729 W/cm^2^ and an open-circuit voltage of 0.91 V, significantly higher than control membranes (Figure 5). The membrane also delivered a specific capacitance of 387 F/g at 1 A/g, outperforming its individual components. The covalently crosslinked PABz-co-Cu MOFs-graft-PIDPA membranes exhibit a well-optimized balance of proton transport, mechanical integrity, and electrochemical stability. These structure–property enhancements stem from the designed synergy between the functional moieties and the 3D network, making them ideal for dual application in HT-PEMFCs and energy storage devices.

**Figure 6:**
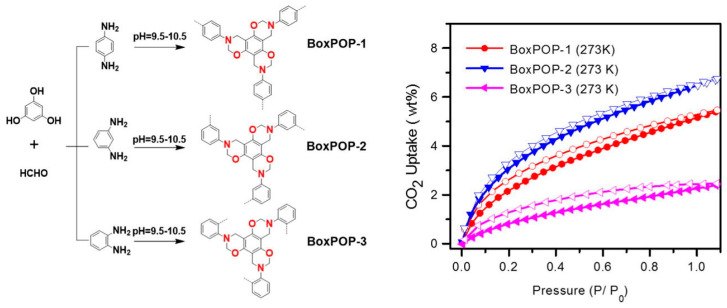
(**Left**) Heteroatom-rich porous organic polymers linked by benzoxazine, and (**Right**) CO_2_ uptake of porous organic polymers. Reprinted with permission from Reference [44]. Copyright 2018 Elsevier.

Xu et al. (2018) [44] developed a novel series of heteroatom-rich porous organic polymers (POPs): BoxPOP-1, BoxPOP-2, and BoxPOP-3, via a Mannich-type condensation reaction involving isomeric diaminobenzenes (p-, m-, o-diaminobenzene), paraformaldehyde, and phloroglucinol to form benzoxazine-linked networks. This strategy forms three benzoxazine rings per phenyl core, integrating nitrogen and oxygen heteroatoms into the polymer backbone without requiring thermal ring-opening polymerization—a key innovation in this work (Figure 6). Structural analysis confirmed successful benzoxazine formation in BoxPOP-1 and BoxPOP-2 with high crosslinking, while BoxPOP-3 showed poor linkage due to steric hindrance from ortho-diaminobenzene, leading to poor polymer formation.

**Figure 7:**
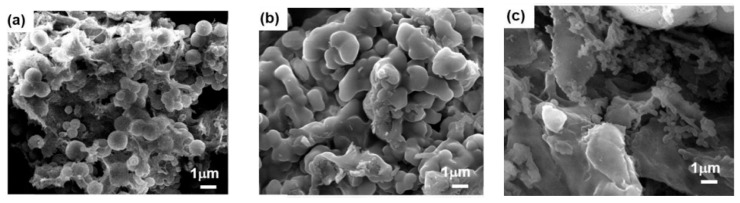
FE-SEM images of (**a**) BoxPOP-1, (**b**) BoxPOP-2, and (**c**) BoxPOP-3. Reprinted with permission from Reference [44]. Copyright 2018 Elsevier.

These structural differences directly influenced the polymers’ porosity (Figure 7) and gas adsorption behavior: BoxPOP-1 and BoxPOP-2 exhibited high surface areas (231 and 225 m^2^/g, respectively) and strong CO_2_ uptake (5.5–6.8 wt.%) with significant isosteric heats of adsorption (27.8–29.8 kJ/mol), attributed to embedded nitrogen and oxygen atoms enhancing CO_2_ affinity. In contrast, BoxPOP-3 had limited porosity and adsorption due to low crosslinking efficiency. Higher surface areas of BoxPOP-1 and BoxPOP-2 correlate with higher CO_2_ adsorption capacities [75,76]. The micropore content is relatively low in all three (BoxPOP-1: 0.0018 cm^3^/g, BoxPOP-2: 0.012 cm^3^/g, and BoxPOP-3: 0.0027 cm^3^/g), suggesting adsorption enhancement is primarily due to chemical affinity rather than physical entrapment. Good thermal stability (up to 250 °C) makes BoxPOP-1 and BoxPOP-2 candidates for real-world CO_2_ capture applications. This structure–property relationship highlights the critical role of monomer geometry in tuning polymer performance for gas capture applications.

**Figure 8:**
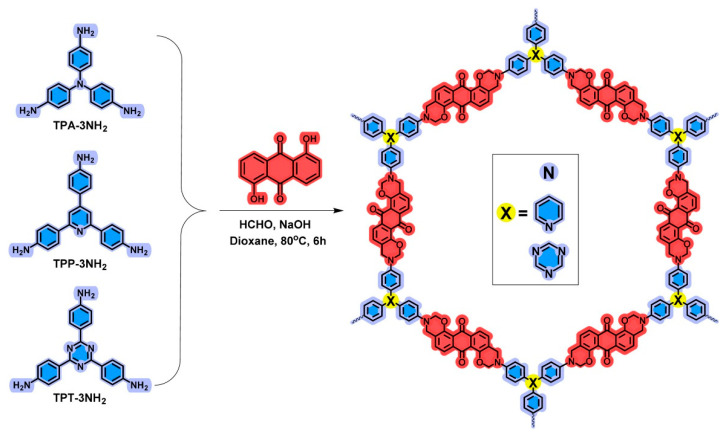
Synthesis of An-TPA, An-TPP, and An-TPT POPs. Reprinted with permission from Reference [27]. Copyright 2025 Elsevier.

In another study, Saber et al. (2025) [27] synthesized a series of heteroatom-rich porous organic polymers via a one-pot Mannich condensation reaction involving 1,5-dihydroxyanthraquinone (An), paraformaldehyde, and various triamines (tris(4-aminophenyl) amine (TPA); 2,4,6-tris(4-aminophenyl)pyridine (TPP); and 2,4,6-tris(4-phenyl)triazine (TPT)) to produce three different benzoxazine monomers—An-TPA, An-TPP, and An-TPT, represented in Figure 8. The resulting benzoxazine-linked frameworks incorporated oxygen and nitrogen heteroatoms into their structures, which were confirmed using FTIR and solid-state ^13^C-NMR. The structure of each polymer was directly influenced by the choice of triamine monomer: An-TPA, derived from tris(4-aminophenyl)amine, exhibited the highest surface area (26.51 m^2^/g), best microporosity (1.96 nm), and greatest electrochemical performance with a specific capacitance of 117.7 F/g at 1 A/g. This superior performance is attributed to its optimal porosity, morphology, and wettability, which enabled better ion transport and pseudocapacitive behavior [77,78]. The pseudocapacitance arises due to faradaic redox reactions involving nitrogen and oxygen heteroatoms embedded in the benzoxazine framework. These atoms provide active sites for fast surface or near-surface redox reactions, contributing to the total capacitance beyond just ion adsorption (EDLC). In contrast, An-TPP and An-TPT showed lower surface areas and capacitance due to planar, rigid structures that limited porosity and surface accessibility. Despite An-TPT having the highest nitrogen content and capacitive contribution, its overall performance was limited by reduced surface area. The impact of monomer geometry, heteroatom accessibility, and polymer morphology on electrochemical efficiency, establishing benzoxazine-linked POPs as promising materials for high-performance supercapacitors.

The work by Tian et al. (2025) [79] presents a strategically engineered sulfonated polybenzoxazine-based membrane (PUBZ) tailored for proton exchange membrane water electrolysis (PEMWE). The synthesis was designed as a three-stage process to incorporate functional sulfonic acid groups (-SO_3_H) and urethane crosslinks into a benzoxazine network. In the first step, a sulfonated benzoxazine monomer (SBZ) was synthesized using a Mannich condensation reaction involving sodium 4-hydroxybenzenesulfonate, 2-(2-aminoethoxy)ethanol, and paraformaldehyde. This monomer, rich in –SO_3_H groups and hydroxyl functionalities, was then subjected to a thermal ring-opening polymerization, yielding a linear oligomer (O-SBZ). In the third step, the oligomer was crosslinked via urethane linkages using hexamethylene diisocyanate (HDI), producing a series of membranes (PUBZ40% to PUBZ70%) with different crosslinking densities based on the monomer-to-crosslinker molar ratio. FTIR and NMR confirmed the successful formation of the benzoxazine and urethane linkages, while SEM and AFM showed homogeneous and dense morphologies. The amorphous nature of the membranes (XRD) aided in flexibility and suppressed crystallinity-related brittleness. Thermogravimetric analysis indicated high thermal stability with two-stage degradation: one from residual hydroxyl and Mannich bridges and the other from aliphatic and aromatic segments. The glass transition temperatures (T_g_) increased with crosslinking (from 40 °C to 65 °C), ensuring membrane resilience at 80 °C operation.

**Figure 9:**
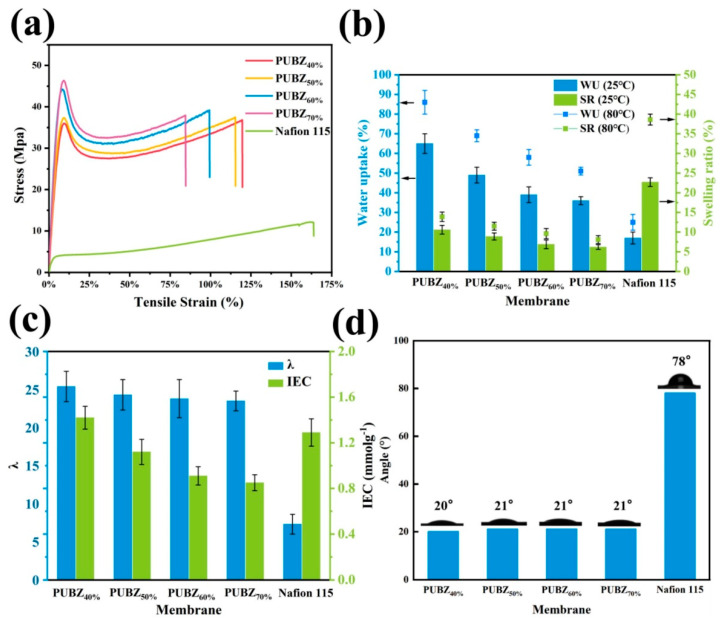
(**a**) Tensile stress–strain curves, (**b**) WU and SR in 25 °C and 80 °C, (**c**) λ and IEC, and (**d**) Water contact angle. Reprinted with permission from Reference [79]. Copyright 2025 Elsevier.

The study details how crosslinking density affects membrane microstructure and electrochemical behavior. Lower crosslinking degrees (PUBZ40% and PUBZ50%) allowed for larger, well-connected hydrophilic domains, promoting high water uptake (up to 86% at 80 °C), high ion exchange capacity (IEC) (1.42 mmol/g), and superior proton conductivity (128 mS/cm for PUBZ40% at 80 °C), as depicted in Figure 9. These features also translated into excellent performance in PEMWE, where PUBZ40% achieved 1.29 A/cm^2^ at 1.95 V, outperforming Nafion 115 (1.02 A/cm^2^). Conversely, increasing the crosslinking density (PUBZ60% and PUBZ70%) led to tighter polymer networks, which reduced the size and connectivity of water channels, decreasing proton mobility but improving mechanical strength, dimensional stability, and hydrogen crossover resistance (down to 536 ppm H_2_ in O_2_ for PUBZ70%) [80,81]. The study demonstrates a precise molecular design strategy, where sulfonic acid functionalization and urethane crosslinking collaboratively control ion transport, dimensional stability, gas barrier properties, and durability. PUBZ40% stands out as the optimal formulation with a balanced trade-off between ionic conductivity and mechanical robustness, positioning it as a viable alternative to costly fluorinated membranes like Nafion in electrolysis applications.

## 4. Tailored Porosity and Surface Area in PBz-Derived Carbons for Enhanced Electrochemical Activity

The study by Liu Wan et al. (2017) [1] introduces a novel method to synthesize graphene oxide/nitrogen-containing porous carbon (GO/NC) nanocomposites by integrating graphene oxide (GO) with polybenzoxazine (PBZ) through in situ ring-opening polymerization, followed by KOH activation. The resulting materials are tailored for high-performance supercapacitor applications. A new bifunctional benzoxazine monomer is synthesized from phenolphthalein, urea, and formaldehyde. GO is incorporated through hydrogen bonding and covalent interactions with PBZ, and the composite is activated with KOH at 700 °C. The nanocomposites exhibit high surface areas (up to 1493 m^2^/g), well-developed hierarchical pore structures (micropores, mesopores, and macropores), enhanced electrical conductivity (up to 11.73 S/cm), and high nitrogen and oxygen content due to the PBZ precursor and GO functional groups [82,83]. The optimal nanocomposite (GO/NC-2 with 1.29 wt.% GO) achieved a specific capacitance of 405.6 F/g at 1 A/g in 6 M KOH. Pyrrolic and pyridinic nitrogen significantly enhance pseudocapacitance in carbon-based electrodes. GO/NC nanocomposites showed CV curves with rectangular shapes and minor Faradaic peaks, and GCD curves with slightly curved triangles—both indicating combined EDLC and pseudocapacitance. This arises from redox reactions involving pyrrolic, pyridinic, and quinone groups in alkaline electrolytes. Excellent rate capability (267.8 F/g at 40 A/g) and cycling stability (95.8% retention over 5000 cycles) were also observed. In symmetric two-electrode systems (1 M Na_2_SO_4_), the device operated at 1.8 V and demonstrated a high energy density of 38.6 Wh/kg at 180 W/kg, maintaining 19.9 Wh/kg at 32.4 kW/kg. The work confirms that introducing a small amount of GO into nitrogen-rich PBZ-derived porous carbon significantly enhances the textural, compositional, and electrical properties of the resulting material. These GO/NC composites display exceptional performance as supercapacitor electrodes due to their optimized porosity, high conductivity, and functionalized surface chemistry, making them strong candidates for future energy storage technologies.

Thirukumaran et al. (2018) [33] synthesized a novel benzoxazine monomer (EM-Bz) from eugenol, melamine, and paraformaldehyde via a solution condensation reaction. The EM-Bz monomer underwent thermal ring-opening polymerization at staged temperatures (100–250 °C) to form polybenzoxazine (PBz). The PBz was then carbonized at 600 °C under a nitrogen atmosphere to form carbonaceous material. The carbonized PBz was chemically activated using KOH at 600 °C, to produce nitrogen-rich carbon sheet (N-CSs) with developed porosity and enhanced surface properties. FT-IR and NMR analyses confirmed the presence of benzoxazine ring structures and nitrogen functionalities. NMR peaks indicated oxazine ring protons and methoxy/allyl groups from eugenol. FESEM images revealed sheet-like morphologies composed of spherical nanoparticles (~20 nm) with hollow interiors, attributed to KOH etching. XPS confirmed nitrogen doping with functional groups like pyridinic N, pyrrolic N, and N-oxide, essential for electrocatalytic activity. XRD patterns showed broad peaks at ~25° and 43°, indicating amorphous to moderately graphitized carbon. Raman spectra had distinct D (1364 cm^−1^) and G (1582 cm^−1^) bands with an I_D/I_G ratio of 1.02, confirming a balance between disorder and graphitic domains. The electrocatalytic analysis showed that N-CSs exhibited an onset potential of −10 mV (vs. RHE) and a Tafel slope of 45 mV/dec, comparable to Pt (34 mV/dec), indicating rapid HER kinetics. Electrochemical impedance spectroscopy (EIS) showed low charge-transfer resistance (R_ct_) with increasing overpotential. Excellent durability and morphological stability were observed after prolonged HER cycling. The synthesis method effectively translates the molecular architecture of polybenzoxazine into nitrogen-doped carbon sheets with high surface area, tailored nitrogen functionalities, hierarchical porosity, and excellent electronic conductivity. These structural features result in outstanding hydrogen evolution reaction (HER) performance, demonstrating the potential of bio-based polybenzoxazine-derived carbon as a low-cost, efficient electrocatalyst.

Barwe et al. (2019) [15] explored the development of highly active and stable bifunctional oxygen electrocatalysts based on cobalt boride (CoₓB) and cobalt phosphide (CoₓP) embedded in polybenzoxazine (pBO)-derived nitrogen-doped carbon (NC) matrices. A low-cost, effective, and durable bifunctional electrocatalyst for both the oxygen reduction reaction (ORR) and the oxygen evolution reaction (OER), which are crucial for applications such as rechargeable metal–air batteries, has been developed. Cobalt boride and phosphide nanoparticles were synthesized from their precursors, cobalt(II)chloride hexahydrate and dichlorobis(triphenylphosphine)cobalt(II), by reductive thermal decomposition. A benzoxazine oligomer (BA-tepa) was prepared from bisphenol A, tetraethylenepentaamine, and formaldehyde. The synthesized catalyst particles were dispersed in BA-tepa and drop-coated onto glassy carbon electrodes. The coated electrodes underwent sequential polymerization and pyrolysis (500–700 °C) in an argon atmosphere to form Co_x_Y/NC composites (Y = B or P). XPS, XRD, SEM, TEM, EDS, and electrochemical techniques were employed to analyze composition, structure, morphology, and electrocatalytic performance. The pyrolyzed composites contain cobalt-based nanoparticles embedded in a nitrogen-doped carbon matrix derived from polybenzoxazine. The composites consist of partially graphitized carbon with amorphous and crystalline cobalt boride/phosphide phases. N-doping introduced pyridinic, pyrrolic, and graphitic nitrogen functionalities that enhance catalytic activity.

**Figure 10:**
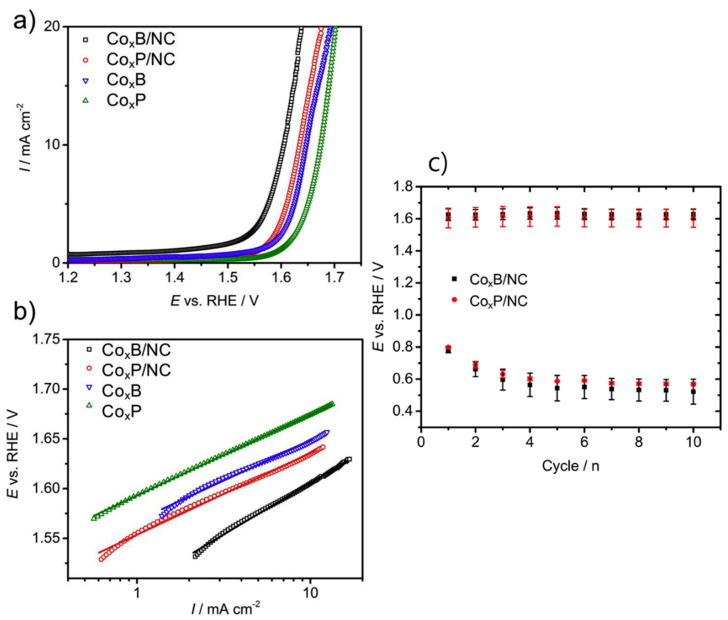
(**a**) LSVs for the OER on Co_x_B/NC, Co_x_P/NC, Co_x_B, and Co_x_P (1600 rpm, O_2_-saturated 0.1 M KOH, 5 mV s^−1^), (**b**) their corresponding Tafel plots, and (**c**) alternating pulse chronopotentiometry for bifunctional stability test. Each cycle consists of 15 min OER at 10 mA cm^−2^ and 15 min ORR at −1 mA cm^−2^ (1600 rpm, O_2_-saturated 0.1 M KOH). Reprinted with permission from Reference [15]. Copyright 2019 Elsevier.

The electrochemical studies showed that both Co_x_B/NC and Co_x_P/NC demonstrated high ORR and OER activities in alkaline media (0.1 M KOH). ORR proceeded predominantly through the 4-electron pathway [84,85], avoiding harmful H_2_O_2_ formation (Figure 10). A low round-trip voltage of ~0.81 V between ORR and OER current densities (−1 and +10 mA/cm^2^, respectively) was achieved. Performance of the synthesized catalysts was comparable to or better than commercial catalysts like RuO_2_ and IrO_2_ for OER. The stability measurements carried out using alternating chronopotentiometry showed good durability, though some degradation in ORR performance occurred due to oxidative deactivation of active sites under OER conditions. This study demonstrates a facile and scalable strategy for preparing high-performance bifunctional oxygen electrocatalysts by integrating cobalt metalloids with a polybenzoxazine-derived nitrogen-doped carbon matrix. The approach not only enhances catalytic activity but also ensures good stability and conductivity without the need for conventional binders like Nafion. This makes the materials promising candidates for energy conversion and storage devices such as rechargeable metal–air batteries.

Later on, Danea Medina et al. (2019) [86] synthesized and optimized Co/Co-Fe nanoparticles embedded in a nitrogen-doped carbon matrix (Co/Co_x_Fe_y_/NC) derived from polybenzoxazine and CoFe layered double hydroxide (LDH) for use as bifunctional oxygen electrocatalysts. The synthesis strategy involves the synthesis of CoFe LDH via co-precipitation and the synthesis of BA-tepa, a nitrogen-rich polybenzoxazine, from bisphenol A, tetraethylenepentaamine, and formaldehyde. The composite was produced by combining CoFe LDH with BA-tepa in different ratios and drop-cast onto glassy carbon electrodes. The mixture underwent stepwise thermal treatment—polymerization (180–200 °C) and pyrolysis (800–1000 °C)—in either an argon or ammonia atmosphere. The samples were denoted as Co/Co_x_Fe_y_/NC-Ar (pyrolyzed in Ar) and Co/Co_x_Fe_y_/NC-NH_3_ (pyrolyzed in NH_3_). XRD and XPS confirmed the formation of metallic Co, Co-Fe alloys, and nitrogen-doped carbon matrices. BET analysis showed higher surface area and porosity for the NH_3_-pyrolyzed sample (139 m^2^/g) compared to the Ar-pyrolyzed sample (93 m^2^/g). SEM and XPS revealed smaller nanoparticles and higher nitrogen content in NH_3_-treated samples. The electrochemical measurements showed that Co/CoxFey/NC-NH_3_ achieved an ORR onset potential of 0.82 V vs. RHE, OER potential of 1.59 V vs. RHE (at 10 mA/cm^2^). Low round-trip voltage of 0.77 V (ORR at −1 mA/cm^2^ to OER at +10 mA/cm^2^) was obtained. Electron transfer number for ORR was found to be between 3.7 and 3.9 (a favorable four-electron pathway). H_2_O_2_ yield significantly reduced in NH_3_ sample (17–22%). Excellent stability was obtained for both ORR and OER under continuous operation. Alternating ORR/OER cycling showed only moderate degradation (~22.5% increase in potential gap). The authors successfully developed a highly effective, stable, and low-cost bifunctional catalyst system using polybenzoxazine-derived nitrogen-doped carbon and CoFe LDH precursors. Pyrolysis in an ammonia atmosphere significantly improved both structural properties and electrocatalytic activity. The optimized materials are promising alternatives to precious metal catalysts for next-generation metal–air batteries and fuel cells.

**Figure 11:**
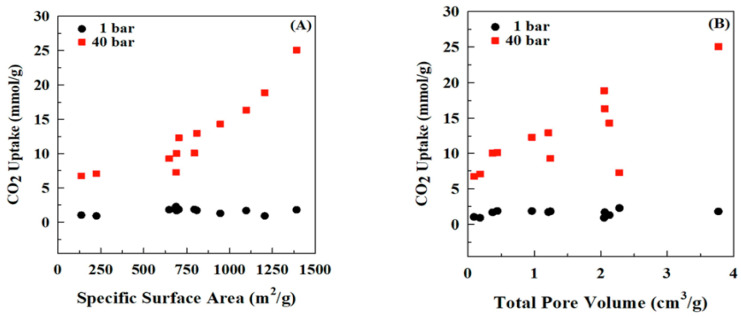
(**A**) Effect of specific surface area and (**B**) pore volume on CO_2_ adsorption at 1 and 40 bar. Reprinted with permission from Reference [46]. Copyright 2023 Elsevier.

Thubsuang et al. (2023) [46] explored the development of nitrogen-rich, ordered-interconnected porous carbon materials for effective CO_2_ capture under high-pressure conditions. Using polybenzoxazine (PBZ) as the carbon and nitrogen source and colloidal silica nanoparticles as templates, the researchers synthesized porous carbon with tunable mesoporous structures. The materials were subjected to carbonization and CO_2_ activation, resulting in exceptionally high surface areas and pore volumes. Among the prepared samples, the activated carbon AC40%Si-800 (prepared with 40 wt.% silica, carbonized at 800 °C, and activated at 900 °C) exhibited the highest CO_2_ adsorption capacity of 25.07 mmol/g at 40 bar and 30 °C (Figure 11), outperforming many other reported materials. Computational studies revealed that only pyridinic nitrogen (N-6) functionalities chemically interacted with CO_2_ via Lewis acid–base interactions, enhancing the adsorption beyond physical means. The findings demonstrate that a combination of high surface area, mesoporosity, and chemically active nitrogen groups is critical for optimizing CO_2_ capture, particularly under high-pressure industrial conditions such as pre-combustion and natural gas treatment processes [87,88].

The material also showed reasonable recyclability, though a decline in performance over cycles was noted, attributed to strong chemisorption and structural pore effects. Overall, this work provides valuable insights into the rational design of high-performance CO_2_ adsorbents using PBZ-derived nitrogen-doped porous carbon frameworks. Table 1 denotes the summary of the performance of PBz composites for energy applications.

## 5. Toughening Strategies for PBz Composites

The following toughening strategies are employed to improve the mechanical robustness of PBz composites, especially addressing their inherent brittleness. Blending PBz with ductile polymers (e.g., polyethersulfone, polyurethane, epoxy resins) helps reduce brittleness [8,14,53,68]. These blends form semi-interpenetrating polymer networks (semi-IPNs), enhancing toughness while maintaining thermal stability. Incorporation of liquid rubber (e.g., carboxyl-terminated butadiene acrylonitrile—CTBN) improves energy dissipation under stress. Rubber domains induce phase separation, creating crack-deflecting structures that resist fracture. The addition of nano-reinforcements (e.g., graphene oxide, carbon nanotubes, nanoclays, silica nanoparticles) significantly enhances fracture toughness and flexural strength [64,69]. These fillers can improve both mechanical integrity and electrochemical functionality. The synthesis of PBz with flexible or aliphatic segments introduces chain mobility, improving ductility. This strategy maintains the thermal properties of PBz while boosting its impact resistance. Hyperbranched polymers (HBPs) incorporated into the PBz network enhance energy absorption capacity and reduce crack propagation. HBPs also lower the curing temperature and improve processability. Introduction of thermoplastics (e.g., polyetherimide, polycarbonate) can create phase-separated domains, promoting plastic deformation and crack blunting under stress [8,67,68]. Modifying the benzoxazine monomers with flexible linkages or bulky side groups reduces crosslink density, improving toughness without compromising thermal stability.

## 6. Conclusions, Key Findings, and Future Perspectives

PBz exhibits a unique combination of thermal stability, chemical resistance, low water absorption, and design flexibility, making it an excellent matrix for conducting composites in energy applications. Incorporating conductive fillers such as graphene, carbon nanotubes (CNTs), Ketjen black, conducting polymers (e.g., polyaniline, polypyrrole), and metal nanoparticles into PBz significantly enhances electrical conductivity and forms percolated conductive networks. Techniques such as drop casting, solution immersion, and thermal curing enable easy fabrication of PBz-based composites with tunable surface roughness, hydrophobicity, and conductivity. Tailoring the monomer structure and polymerization conditions (e.g., temperature, curing agents) directly affects mechanical, thermal, and electrical performance. For instance, hyperbranched ionic liquids can reduce curing temperatures and increase toughness. PBz composites demonstrated high specific capacitance (up to 387 F/g) for supercapacitors. PBz-derived nitrogen-doped carbons showed excellent performance in electrocatalysis (HER, ORR, OER), comparable to Pt or RuO_2_/IrO_2_. Composites used in proton exchange membranes showed high proton conductivity (up to 128 mS/cm) and superior mechanical and oxidative stability. Nitrogen-rich porous carbons derived from PBz with tailored pore structures achieved high CO_2_ adsorption capacity (25.07 mmol/g) under high-pressure conditions, driven by pyridinic nitrogen functionalities. Use of bio-based monomers (e.g., eugenol) and scalable methods like template-assisted synthesis and KOH activation supports eco-friendly and cost-effective fabrication of advanced functional materials. Synergistic effects from PBz-metal, PBz-MOF, and PBz-polymer systems lead to enhanced electrochemical and mechanical properties, crucial for dual-functionality in energy storage and conversion. These findings collectively demonstrate that PBz-based conducting materials are highly adaptable, scalable, and tunable platforms suited for advanced supercapacitors, electrocatalysts, fuel cells, and CO_2_ capture systems.

The challenges faced by polybenzoxazine, particularly in energy-related applications, include the following: *low electrical conductivity*—pristine PBZs are electrically insulating, limiting their direct application in electrochemical devices like supercapacitors and batteries. This requires modification via incorporation of conductive fillers like graphene, carbon nanotubes, and metal oxides; *limited porosity*—PBZ polymers often suffer from low inherent porosity, reducing ion-accessible surface area critical for electrochemical energy storage. Achieving hierarchical porosity through templating or chemical activation is often necessary; *processing difficulties*—The high curing temperature (typically above 200 °C) required for ring-opening polymerization of benzoxazine monomers can be energy-intensive and processing-restrictive. This can lead to incomplete polymerization and affect mechanical properties if not properly managed; *brittleness*—PBZs exhibit brittle mechanical behavior post-curing due to their rigid crosslinked network. This brittleness restricts their use in flexible or wearable electronics unless blended or copolymerized with ductile materials; *poor solubility of precursors*—benzoxazine monomers and resulting polymers can have limited solubility in common solvents, complicating solution processing and film fabrication; *cycling stability*—in supercapacitor applications, PBZ-based electrodes sometimes exhibit capacity fading during repeated charge/discharge cycles, especially if not well-stabilized or hybridized; and *cost and synthesis complexity*—the synthesis of functional benzoxazine monomers often involves multi-step reactions, increasing costs and time, especially for those with heteroatom-rich structures designed for pseudocapacitive behavior.

Looking forward, there is vast potential for designing multifunctional PBz composites through molecular engineering and advanced fabrication techniques such as 3D printing, atomic layer deposition, and interface modulation. Future research should prioritize enhancing long-term operational stability, reducing synthesis cost, and developing environmentally friendly routes. Additionally, leveraging bio-based benzoxazine precursors and exploring green activation methods can further promote sustainability. The integration of PBz-based systems into real-world devices such as flexible electronics, wearable sensors, and hybrid batteries will require interdisciplinary collaboration and scale-up studies. With continued innovation in materials chemistry and device engineering, PBz-based conducting materials are poised to play a pivotal role in the transition to clean and efficient energy technologies.

## Figures and Tables

**Table 1:** Summary of performance metrics of PBz-based composites for energy applications.

Material	Composition	Performance	Application
KB-PBZ Composite [6] (Bisphenol-A based Bzo)	Ketjen Black + PBz	WCA ≈ 160°, SA ≈ 3°, Conductive surface	Supercapacitor/ Superhydrophobic Coating
MWNTs/PBZ [61] (Bzo with carbonitrile group)	MWNTs in BOZ matrix, 20 immersion cycles	WCA up to 152°, SA ≈ 3°, Sheet resistance: 3.41 × 10^3^ Ω/sq	Superhydrophobic and Conductive Textile
HBP-AMIM^+^PF_6_^−^/BA-ECC [8] (Bisphenol-A based Bzo)	Hyperbranched Ionic Liquid + Benzoxazine/Epoxy	Tensile strength ↑ 76.6%, Impact strength ↑ 80.4%, Tg ↑, T_5_% ↑ 30 °C	Structural Composite
Graphene/Graphite/CNT-PBZ [41] (Bisphenol-A based Bzo)	7.5% graphene + variable CNT/graphite	Conductivity: 364 S/cm, Thermal conductivity: 21.3 W/m·K, Flexural strength: 41.5 MPa	PEMFC Bipolar Plate
PBA/EG Multilayer BP [71] (Bisphenol-A based Bzo)	Graphite-PBA-Graphite layers	In-plane conductivity: 278.85 S/cm, Flexural strength: 75.75 MPa, WCA ≈ 99°–102°, ASR: 9.70 mΩ·cm^2^	PEMFC
PABz-co-Cu MOFs-graft-PIDPA [53] (Bzo from ethylene diamine and 5-sulphosalicylic acid)	MOF-linked benzoxazine membrane	Proton conductivity: 0.0757 S/cm, Capacitance: 387 F/g, Tensile strength: 3.87 MPa	HT-PEMFC & Supercapacitor
BoxPOP-1/2 [44] (Bzo from diaminobenzenes and phloroglucinol)	Benzoxazine-linked porous polymers	CO_2_ uptake: 5.5–6.8 wt.%, Surface area: 225–231 m^2^/g, Qst: 27.8–29.8 kJ/mol	CO_2_ Capture
An-TPA POP [27] (Bzo from 1,5-dihydroxyanthraquinone and triamines)	Benzoxazine with tris(4-aminophenyl) amine	Surface area: 26.51 m^2^/g, Capacitance: 117.7 F/g	Supercapacitor
PUBZ Membrane (40%) [79] (Bzo from sodium 4-hydroxybenzenesulfonate and 2-(2-aminoethoxy)ethanol)	Sulfonated PBZ + HDI	Proton conductivity: 128 mS/cm @ 80 °C, IEC: 1.42 mmol/g, Peak current density: 1.29 A/cm^2^ @ 1.95 V	PEM Water Electrolysis
GO/NC [1] (Bzo from phenolphthalein and urea)	Graphene oxide + PBz-derived N-carbon	Surface area: 1493 m^2^/g, Conductivity: 11.73 S/cm, Capacitance: 405.6 F/g	Supercapacitor
N-CSs from EM-Bz [33] (Bzo from eugenol and melamine)	Eugenol-melamine derived N-carbon sheets	Onset potential: −10 mV (vs. RHE), Tafel slope: 45 mV/dec	HER Electrocatalyst
CoₓB/NC, CoₓP/NC [15] (Bzo from bisphenol A and tetraethylenepentaamine)	Co-metalloids in PBz-derived N-doped carbon	Round-trip voltage: 0.81 V, Good bifunctional stability	ORR/OER Electrocatalyst
Co/CoxFey/NC-NH_3_ [88] (Bzo from bisphenol A and tetraethylenepentaamine)	CoFe LDH + BA-tepa PBz	Onset potential (ORR): 0.82 V, OER: 1.59 V, Round-trip: 0.77 V	ORR/OER Catalyst
AC40%Si-800 [46] (Bzo from bisphenol A and Tetraethylenepentaamine)	PBz-derived porous carbon with silica template	CO_2_ uptake: 25.07 mmol/g @ 40 bar, 30 °C	CO_2_ Capture

↑ The symbol means an increase in the property.
